# Polyvinyl Alcohol Microspheres Reinforced Thermoplastic Starch Composites

**DOI:** 10.3390/ma11040640

**Published:** 2018-04-21

**Authors:** Bin Guo, Dongdong Zha, Bengang Li, Peng Yin, Panxin Li

**Affiliations:** 1 College of Science, Nanjing Forestry University, Nanjing 210037, China; 15062225802@163.com (D.Z.); 15651851089@163.com (B.L.); yinpeng416@163.com (P.Y.); 2 Agricultural and Forest Products Processing Academician Workstation of Henan Province, Luohe 462600, China; 13951778290@163.com; 3 Post-Doctoral Research Center of Henan Nanjiecun Group, Luohe 462600, China

**Keywords:** polyvinyl alcohol microspheres, thermoplastic starch, composites

## Abstract

We reported a new method to prepare polyvinyl alcohol (PVA)/thermoplastic starch (TPS) composites by using polyvinyl alcohol microspheres (PVAMS). The PVAMS/TPS composites were characterized using tensile test, scanning electron microscopy (SEM), dynamic mechanical thermal analysis (DMTA) and thermogravimetric analysis (TGA). The results exhibited that adding small amounts of PVAMSs can effectively improve the mechanical strength and toughness of the composites, especially for the 1 wt %PVAMS in TPS matrix, with a tensile strength of 3.5 MPa, an elongation at break at 71.73% and an impact strength of 33.4 kJ/m^2^. Furthermore, the SEM and shift in the tan δ peak (T_α_ and T_β_) at the maximum value of 69.87 and −36.52 °C indicates that the PVAMS decreased the mobility of the amorphous starch molecules due to the strong intermolecular hydrogen bonds between PVAMS and TPS. The peak temperature of maximum decomposition rate (Tp) of 1 wt % PVAMS/TPS composites increased about 5 °C compared with TPS in TGA curves.

## 1. Introduction

The development of thermoplastic starch (TPS)has received considerable attention over the last two decades due to its biodegradability, renewability and low cost. However, the retrogradation and relatively low mechanical properties of TPS, particularly in wet and dry environments, still significantly restrict their further application [1,2], even though TPS has considerable market prospects.

In order to enhance the performance of TPS, three main strategies have been applied in recent decades, namely,(1) chemical modification of starch (e.g., acetylation, oxidation) [3,4]; (2) blending of TPS with polymers such as polyvinyl alcohol (PVA) [5], polyethylene [6], polylactic acid [7], and poly(butylene adipate-co-terephthalate) [8]; and (3) addition of fibers, including natural fibers (cotton [9], flax [10], sisal [11] and pea fibers [12]), synthetic fibers [13,14], and micro/nanofibers [15,16] as reinforcement for TPS.

Among these strategies, blending TPS and PVA is a good choice because both are environmentally friendly and biodegradable materials [17,18] (e.g., the well-known industrial product Mater-Bi from Novamontin Italy). Currently, researchers primarily focus on the effects of plasticizers [19,20] or cross-linking agents [21] on the mechanical properties of TPS/PVA. However, the PVA in the above studies was at least 40–50 wt %. This high PVA content would translate to high cost and price for the TPS/PVA blends, rendering them unsuitable for further application. To reduce the usage of PVA, and inspired by glass bead-filled polypropylene composites [22], which can improve the stiffness and toughness of PP composites, we propose to use polyvinyl alcohol microspheres (PVAMSs), the particle morphology of PVA, to reinforce the TPS matrix.

In this work, we report the new TPS/PVA composite by adding a small amount of PVAMSs to a TPS matrix for the first time, and we focus on the effect of PVAMS on mechanical properties, fracture morphology, dynamic mechanical properties and thermal stability for the PVAMS-reinforced TPS composites.

## 2. Experimental Section

### 2.1. Materials

The corn starch (13.6 wt % moisture content) used in this study was of food grade and was supplied by the Shandong Hengren Industry and Trade Company (Zaozhuang, China). Glycerol (CP) and polyvinyl alcohol (PVA, PD = 1750 ± 50; Shanghai, China) were purchased from the Sinopharm Chemical Reagent Co., Ltd. (Shanghai, China).

### 2.2. Preparation of PVAMS/TPS Composites

PVAMSs were prepared according to the procedure outlined in [23]. Sorbitan monostearate (0.5 g) as a dispersant was dissolved in 40 mL liquid paraffin to form a continuous oil phase in a round-bottom flask. The PVA solution (5 wt %, 20 mL) and 5mL glutaraldehyde were mixed to form a dispersed water phase, followed by the addition of 2 mL hydrochloric acid (1 mol/L) as a catalyst. The mixture was stirred to disperse the catalyst thoroughly, resulting in a reversed-phase suspension system. PVAMSs were formed during this reverse suspension cross-linking reaction, which was carried out at 50 °C for 1.5 h with appropriate mechanical stirring. The resultant microspheres were thoroughly washed with N-heptane and distilled water and dried under vacuum in a drying oven at 60 °C for 24 h.

Corn starch here was not dried before processing although the water content of starch highly influences the properties of TPS [24]. It was manually premixed at a ratio of 3:1 with glycerol in polyethylene bags and stored overnight. After this preliminary step, the mixture was further processed by adding a corresponding amount of PVAMS and blending at 25,000 rpm for 15 s using a high-speed mixer. The PVAMS loading (0, 0.5, 1, 1.5, 2, 3 wt %) was based on the amount of TPS. The mixtures were fed into a twin-screw plastic extruder (SHJ20, Nanjing Giant Machinery Co., Ltd., Nanjing, China) operating at 150 rpm. The temperature profile along the extruder barrel was based on four heating zones: 115, 120, 125, and 115 °C. The dumbbell-shaped specimens (2 mm thick) of the TPS and composites were prepared directly by injection molding machine (BV90, APOLLO, Shanghai century-win mechanical industry Co., Ltd., shanghai, China), in which the melt temperature was 145 °C, and the mold temperature was about 20~30 °C, the injection pressure was 55 MPa, holding pressure was 40 MPa, backpressure was 5 MPa and cooling time was 40 s.

### 2.3. Characterization of Composites

After processing, the specimens of the TPS and composites were put into some polyethylene bags immediately to avoid moisture uptake and retrogradation, and then stored in an oven at 25 °C for 24 h.

Then the tensile tests were performed at room temperature in accordance with the ASTM D638 standard on a testing machine (SANS, MTS Systems Corporation, Shenzhen, China). Five to eight specimens were tested for each sample, and the average values of the measured properties were reported. Impact measurements were conducted in accordance with the ASTM D256-10 standard on a testing machine (XJC-25D, Chengde precision testing machine Co., Ltd., Chengde, China). Impact tests using impact energy of 7.5 J were conducted at an impact rate of 3.8 m/s. At least five specimens were tested for each formulation.

The fractured surfaces of the composites were studied using an environmental scanning electron microscope (SEM, Quanta 200, FEI Company, Hillsboro, OR, USA). The samples were obtained from the dumbbell-shaped specimens after the tensile measurements. The fractured part of specimen was cut into10 mm × 5 mm × 4 mm near the fracture surface. The fractured faces were vacuum-coated with gold prior to analysis, and the tungsten filament was operated at 20 kV.

Dynamic Mechanical Thermal Analysis (DMTA) were performed using a Netzsch 242E instrument (NETZSCH-Gerätebau GmbH, SELB, Bavaria, Germany) in three-point bending mode at a frequency of 5 Hz, corresponding to a maximum displacement amplitude of 60 μm. The temperature ranged from −120 to 120 °C, and the heating rate was 3 °C/min. The dimensions of the specimens were 50 mm × 10 mm × 4 mm. At least three specimens were replicated for each sample.

Thermal stability curves of the samples were recorded on a thermogravimetric analyzer (TG 209 F1, Netzsch, Germany). The samples were analyzed under a nitrogen atmosphere over a temperature range of 25–600 °C at a heating rate of 20 °C/min.

## 3. Results and Discussion

### 3.1. Mechanical Properties

**Figure 1:**
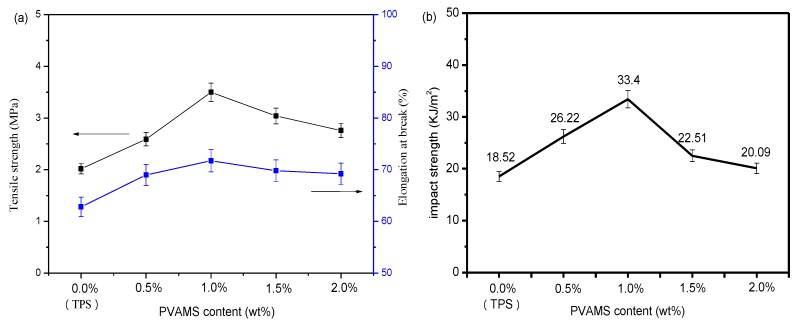
Mechanical properties of thermoplastic starch (TPS) and polyvinyl alcohol microspheres (PVAMS)/TPS composites. (**a**) Tensile strength(in black) and elongation at break(in blue); (**b**) impact strength.

Figure 1a shows the tensile strength and elongation at break of TPS were about 2.02 MPa and 62.82% respectively. After adding a small amount of PVAMSs, the tensile strength was obviously increased up to 3.5 MPa for the composite containing 1 wt % PVAMS. This is an indication of the effective reinforcement of PVAMS in TPS matrix, which can be ascribed to the better mechanical properties of the cross-linked PVAMS. In addition, the elongation at break also increased from 62.82 to 71.73% with increasing PVAMS content (0 to 1 wt %), this maybe related to the intermolecular hydrogen-bond interactions between PVAMSs and TPS, in the meanwhile, PVAMSs also can disperse uniformly in the TPS matrix.

Figure 1b displays the impact strength with increased PVAMS contents for the TPS and composites. The results indicate that the impact strength of composites is higher than that of TPS and this implies that the toughness is improved by the presence of PVAMS. The impact strength of composite increased to 33.4 kJ/m^2^ at 1 wt % PVAMS compared to TPS, and this may be related to the craze induced by PVAMS in the matrix around the surface, which can absorb the impact fracture energy effectively [22]. 

### 3.2. Surface Topography

**Figure 2:**
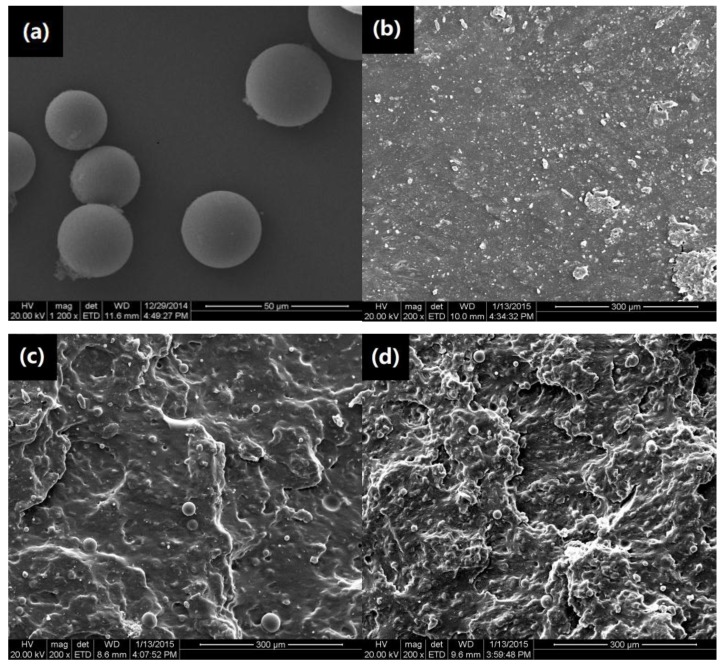
SEM images of PVAMS. (**a**) And the fractured surfaces of PVAMS/TPS composites with PVAMS content of (**b**) 0 (TPS); (**c**) 1; (**d**) 2 wt %.

Figure 2a presents the PVAMS with the diameter about 20 μm. The fractured cross-sections of TPS featured a homogeneously smooth surface in Figure 2b, consistent with literature reports [25]. On the contrary, the fractured surface of PVAMS/TPS composites exhibited different morphologies because of the presence of PVAMS. As observed in Figure 2c, most of the PVAMS was embedded and dispersed uniformly in the fractured surface at 1 wt % PVAMS in TPS matrix as a stable continuous phase. No aggregation was found, suggesting that the PVAMS was dispersed homogeneously in the PVAMS/TPS composites. Stress transfer and absorbance of the impact fracture energy can be achieved effectively, in accordance with the highest tensile and impact strength at 1 wt % PVAMS.

However, the fracture surface became rougher at 2 wt %PVAMS in Figure 2d, and many PVAMSs were observed at the fractured surface, and more possible aggregation occurred in the TPS matrix, suggesting a negative effect on mechanical properties. Namely, the PVAMSs could not transfer stress and absorb impact energy effectively during the tensile and impact process and did not enhance the mechanical properties of the material [12]. This finding is consistent with the results of the mechanical property analysis.

### 3.3. Dynamic Mechanical Thermal Properties 

**Figure 3:**
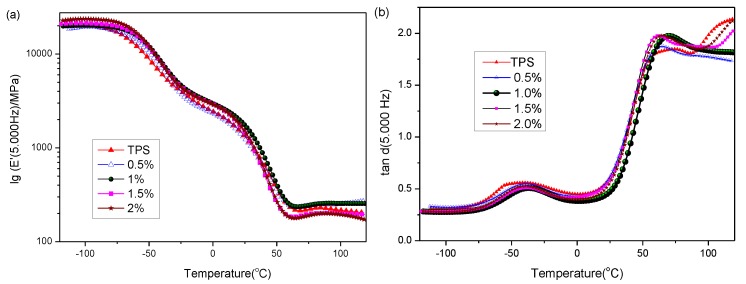
DMA(dynamic mechanical analysis) curves of PVAMS/TPS composites prepared at varying PVAMS content (0–2 wt %). (Frequency: 5 Hz). (**a**) E’ vs. Temp; (**b**) tan δ curve.

Figure 3a shows the storage modulus (E’) curve of TPS and PVAMS/TPS composites as a function of temperature by DMA(dynamic mechanical analysis). In general, the modulus decreased with the increased temperature. In the low-temperature zone, the storage modulus of PVAMS/TPS increased with increasing PVAMS content compared to TPS, evidencing the stiffening effect of the reinforcing PVAMS. In the high-temperature zone, especially after the glass transition temperature, the relative higher storage moduli for 1 wt % PVAMS/TPS indicated the strong interaction between PVAMSs and the TPS matrix, in accordance with the results obtained in mechanical and SEM experiments. 

The effect of temperature and PVAMS content on the phase angle (tan δ) of the composites is plotted in Figure 3b, and tan δ peak temperatures are presented in Table 1. In general, TPS showed biphasic behavior, presenting two different transitions in the tan δ curves. The first low-temperature peak (T_β_), appearing at −40 °C, corresponds to the phase rich in glycerol. This relaxation peak appears more intensely in TPS and composites presenting low PVAMS content. This shift in the tan δ peak is indicative of restrictions to the cooperative motion of the segmental chains of the TPS matrix in the vicinity of the microsphere.

Meanwhile, the second transition assigned to starch occurred at a higher temperature peak (T_α_) of approximately 50 °C, which is the Tg. This is also in accordance with literature reports [26]. According to the tan δ curve and transition temperature in Table 1, the added PVAMS increases the glass transition temperature of the composite material. When PVAMS content was 1%, the maximum transition temperatures (69.87 °C) indicates that PVAMSs inhibit the relaxation process, leading to more rigid systems and consequently increasing the temperature of the glass transition. This behavior can result from a decrease in the mobility of the amorphous starch molecules caused by the strong intermolecular hydrogen bonds between PVAMS and TPS. However, the glass transition temperature decreases with further increasing PVAMS. This may be due to the possible agglomerates of PVAMS in the TPS matrix [27], and another reason may be due to the more PVAMSs dispersed unevenly in TPS, which would destroy the intermolecular hydrogen-bonds interaction between PVAMS and TPS, resulting in the decreased Tg.

### 3.4. Thermal Stability

**Figure 4:**
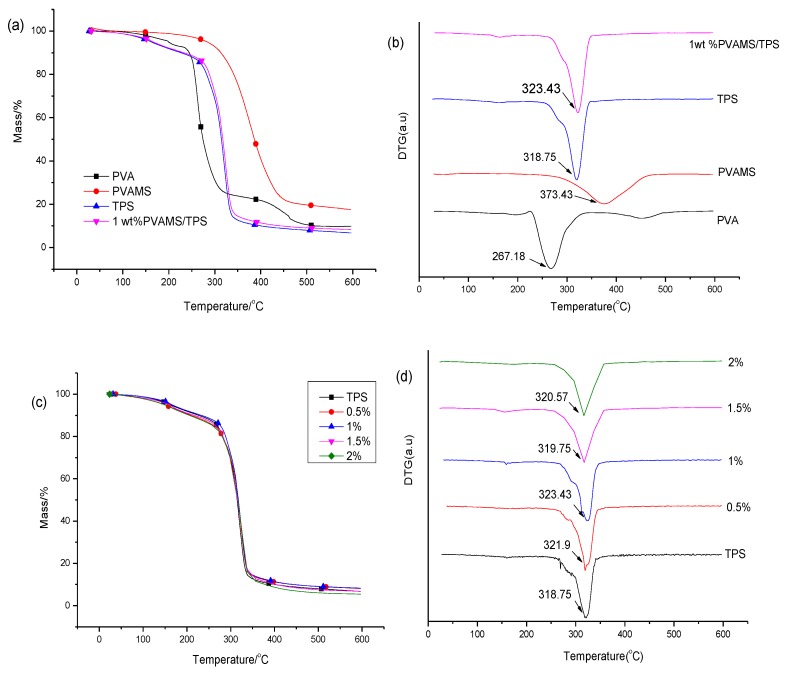
TG(thermogravimetry) (**a**) and DTG(differential thermogravimetry) (**b**) curves forpolyvinyl alcohol(PVA), PVAMS, TPS and 1 wt % PVAMS /TPS composites; TG (**c**) and DTG (**d**) curves of PVAMS/TPS composites prepared with varying PVAMS content (0–2 wt %).

The TG (thermogravimetry)and DTG (differential thermogravimetry) curves for PVA, PVAMS, TPS and 1 wt %PVAMS /TPS composite are shown in Figure 4a,b. PVAMS has better thermal stability with initial decomposition temperatures at approximately 300 °C. Accordingly, the peak temperatures in DTG curves, corresponding to the maximum decomposition rate (Tp), differ significantly. PVAMS have higher Tp (373.43 °C) than PVA (267.18 °C), indicating the better thermal stability of PVAMS due to its cross-linked structure. When 1 wt % PVAMS was added to TPS further, the peak temperature Tp shifted from 318.75 to 323.43 °C. Although this temperature varies by only 5 °C, it is clear that PVAMSs benefit the thermal stability of the PVAMS/TPS composite.

Figure 4c,d shows TG and DTG curves of PVAMS/TPS composites with varying PVAMS content. The thermal weight loss below 150 °C is caused by volatilization of smaller molecules including water and glycerol. Thermal decomposition of PVAMS/TPS began at approximately 300 °C. In DTG curves, the peak temperature corresponded to Tp. We can see the Tp of PVAMS/TPS composites is slightly larger than that of pure TPS (318.75 °C), and the highest value occurs at 323.43 °C for 1 wt % PVAMS in TPS, which is also higher than 320.57 °C for 2 wt % PVAMS/TPS. Although this difference was quite minor, it gives further evidence of the better performance of 1 wt % PVAMS/TPS, consistent with the former results. 

## 4. Conclusions

In this article, PVAMS were used to produce a new PVAMS reinforced TPS composite. From the results obtained, several conclusions can be made. It is shown that good dispersion and interaction with the TPS matrix was achieved especially for the 1 wt % PVAMS, with a tensile strength of 3.5 MPa, an elongation at break at 71.73% and an impact strength of 33.4 kJ/m^2^. It demonstrates that obvious changes in dynamic mechanical properties in glass-transition temperature Tg for the reason that the PVAMS constrained mobility of the chain in TPS matrix. In addition, the increased Tp in DTG proved that the thermal stability of the 1 wt %PVAMS/TPS composite was also improved compared with that of TPS.

## Figures and Tables

**Table 1:** Transition temperature of PVAMS/TPS composites prepared at varying PVAMS content (0–2 wt %). (Frequency: 5 Hz).

	* TPS	0.5%	1%	1.5%	2%
T_β_	−46.71	−40.53	−36.52	−38.27	−39.40
T_α_	64.38	64.61	69.87	62.65	64.02

* TPS is 0 wt % PVAMS/TPS composite.
